# Alcohol marketing in televised international football: frequency analysis

**DOI:** 10.1186/1471-2458-14-473

**Published:** 2014-05-20

**Authors:** Jean Adams, James Coleman, Martin White

**Affiliations:** 1 Institute of Health & Society, Newcastle University, Baddiley-Clark Building, Richardson Road, Newcastle upon Tyne NE2 4HH, UK

**Keywords:** Advertising, Sport, Media

## Abstract

**Background:**

Alcohol marketing includes sponsorship of individuals, organisations and sporting events. Football (soccer) is one of the most popular spectator sports worldwide. No previous studies have quantified the frequency of alcohol marketing in a high profile international football tournament. The aims were to determine: the frequency and nature of visual references to alcohol in a representative sample of EURO2012 matches broadcast in the UK; and if frequency or nature varied between matches broadcast on public service and commercial channels, or between matches that did and did not feature England.

**Methods:**

Eight matches selected by stratified random sampling were recorded. All visual references to alcohol were identified using a tool with high inter-rater reliability.

**Results:**

1846 visual references to alcohol were identified over 1487 minutes of broadcast - an average of 1.24 references per minute. The mean number of references per minute was higher in matches that did vs did not feature England (p = 0.004), but did not differ between matches broadcast on public service vs commercial channels (p = 0.92).

**Conclusions:**

The frequency of visual references to alcohol was universally high and higher in matches featuring the only UK home team - England - suggesting that there may be targeting of particularly highly viewed matches. References were embedded in broadcasts, and not particular to commercial channels including paid-for advertising. New UK codes-of-conduct on alcohol marketing at sporting events will not reduce the level of marketing reported here.

## Background

Alcohol marketing includes not just paid-for advertising, but also a range of other activities including pricing, promotions, branding, and sponsorship of individuals, organisations and cultural and sporting events.

Alcohol marketing is known to effect consumption, particularly in children. Exposure to alcohol marketing decreases the age that adolescents who have not yet begun to consume alcohol begin to drink, and increases the amount that adolescents who already drink consume [1-3]. One arena is which both children and adults are exposed to alcohol marketing is professional sports.

Research from around the world demonstrates the increasingly pervasive nature of professional sports-related alcohol marketing. Sophisticated multi-pronged approaches include naming of competitions, advertising in stadia, branding on players and officials uniforms, on-screen logo placement during television broadcasts, and linked commercials during and around programming segments [4,5]. Given the emotionally captivating nature of professional sports, this embedded linkage of alcohol within televised sport may be more compelling than more traditional television advertising [5]. Recent evidence suggests that celebrity endorsement within food marketing increases the effects on food consumption [6]. Although similar data is not available for alcohol marketing, the association of alcohol marketing with professional sportspeople may potentiate its effects. Embedded marketing in professional sporting events can also allow manufacturers to bypass regulations that tend to focus on traditional advertising.

The effect of marketing is a function of both impact, or creative content, and exposure. In the UK, alcohol marketing is self-regulated by an industry-sponsored code-of-practice [7], including a new code on event sponsorship that came into force in January 2014 [8]. These codes focus on not marketing specifically to children and not associating alcohol with socially successful or anti-social behaviour – that is, the creative content of marketing. Although recent data questions how well these codes are being implemented [9], beyond these restrictions on content, there are no controls on exposure to non-advertising alcohol marketing. Association football, or soccer (referred to as ‘football’ henceforth), is believed to be the world’s most popular spectator sport. Almost half of the global population watched at least one minute of the final match of the last FIFA (Fédération Internationale de Football Association) World Cup championship in South Africa in 2010 [10], with more than 20 million viewers in the UK alone [11].

The UEFA (Union of European Football Associations) European Football Championship is an international football tournament contested every four years by the senior men’s teams of UEFA member countries and is considered the second most prestigious tournament after the FIFA World Cup. The 14th European Football Championships (EURO2012) were held in June-July 2012 in Ukraine and Poland. Sixteen national teams qualified and were drawn into four groups. Round-robin group matches determined progression to the knock-out quarter-finals, semi-finals and final. In total, 31 matches were played, attracting large television audiences and breaking a number of national records for most-viewed programme. In the UK, the television audience for the England vs Italy quarter-final exceeded that for the marriage of the Duke and Duchess of Cambridge [12].

*Carlsberg* was the official beer of EURO2012. Among other things, this entitled *Carlsberg* to: have their logo on pitch side electronic sponsor boards and many other areas; have exclusive marketing rights for alcoholic beverages within stadia and ‘fan zones’ (outdoor areas where fans without match tickets could congregate to watch live matches on giant screens); and use EURO2012 logos on their products [13].

Although previous research has explored the frequency of alcohol marketing in televised soccer [14] rugby [15,16], cricket [5,16], and other sports [16-18], most previous work has focused on domestic competitions without large international audiences. The authors are not aware of any previous work that has explored alcohol marketing in televised, international soccer.

In the UK, EURO2012 live broadcasts were split between a non-commercial public service broadcaster (BBC1) and a commercial broadcaster (ITV1), with each showing 15 pre-final matches and the final simultaneously broadcast on both channels. Both channels are free to view. This provided a unique opportunity to explore the variation in alcohol marketing between non-commercial and commercial channels and thus the proportion of marketing that is truly ‘embedded’ in broadcasts.

Of the UK international football teams (England, Scotland, Wales and Northern Ireland), only England qualified for EURO2012. EURO2012 matches featuring England predictably attracted higher UK television audiences than other matches. This provided a further opportunity to determine if there was any particular effect of expected viewer numbers on alcohol marketing within matches.

### Aims

The aim of this work was to determine the frequency and nature of visual references to alcohol in a representative sample of matches from EURO2012 broadcast in the UK; and establish if the frequency and nature of these varied between matches broadcast on public service and commercial channels, or between matches that did and did not feature England.

## Methods

A frequency analysis of visual references to alcohol was conducted in a representative sample of EURO2012 matches broadcast on UK television.

### Selection of broadcasts

Eight matches were selected using stratified random sampling to reflect the aims of the research. The sample size was a pragmatic decision based on time available. The final sample included four matches broadcast on BBC1 and four on ITV1; all four matches featuring England plus four other matches; and matches representative of each tournament stage – four group stage matches, two quarter-final matches, one semi-final match, and the final (Table 1).

**Table 1:** Characteristics of eight EURO2012 matches broadcast on UK television

	***Ireland v Croatia***	***France v England***	***Sweden v England***	***England v Ukraine***	***Germany v Greece***	***England v Italy***	***Portugal v Spain***	***Spain v Italy***	***Total***
Date	10 June	11 June	15 June	19 June	22 June	24 June	27 June	1 July	--
Kickoff time (CET)^a^	2045	1800	2045	2045	2045	2045	2045	2045	--
Tournament stage	Groups	Groups	Groups	Groups	Quarter final	Quarter final	Semi-final	Final	--
Channel	ITV1	ITV1	BBC1	ITV1	ITV1	BBC1	BBC1	BBC1	--
Broadcast length (mins available)	162	208	136	190	154	227	214	196	1487
UK TV viewers (millions)^b^	6.61	9.88	14.25	12.98	7.05	20.34	10.30	12.41	93.82

^a^CET: Central European Time; ^b^Average audience for full broadcast, provided by British Audience Research Bureau.

Selected live broadcasts were digitally video recorded in their entirety, including: all normal playing time plus any added time, extra time, or penalty shoot-outs; pre- and post-match interviews and discussion; the half-time break; and any commercial breaks.

### Coding framework & outcome measures

A coding framework, used in previous work [14], was used to determine the frequency of visual references to alcohol in included broadcasts. All visual references to alcohol both during programming content and during commercial breaks were included. Visual references to alcohol were defined as identifiable alcohol logos or other branding. Each time a visual reference to alcohol was newly identifiable on-screen it was counted once, irrespective of how long it was on the screen, and whether or not it had been previously counted (e.g. during previous camera shots of the same advertising hoarding). If many visual reference to alcohol were shown in a single shot these were all counted individually; and any visual references to alcohol broadcast repeatedly (e.g. during replays) were re-counted every time they were shown.

All visual references to alcohol were categorised in terms of location, alcohol type and broadcast segment. Location was categorised as ‘pitch-side’ – that is, on or alongside the field of play, ‘interview boards’ – that is backdrops featuring sponsor logos that players, managers, and other staff are interviewed in front of, ‘adverts’ – during commercial breaks, and ‘other’. Alcohol type was categorised as beer, cider, spirits, wine or alcopops. Broadcast segment was categorised as pre-match, first half, half time break, second half, extra-time and penalties, post-match, or commercial breaks. These were collapsed for analysis into ‘in-play’ (first half, second half, and any extra-time and penalties) and ‘out-of-play’ (pre-match, half time break, post-match and commercial breaks.

### Data collection and statistical analysis

Recordings of selected broadcasts (made in 2012) were systematically coded by one researcher (JC; in 2013) using the pause and rewind facility as required. Chi-squared and t-tests were used to compare the frequency and nature of alcohol marketing broadcast on public service and commercial channels, and between matches that did and did not feature England.

The coding framework has previously been reported to have good inter-rater reliability [14]. Inter-rater reliability was assessed in this study by asking a second coder to duplicate code one randomly selected broadcast. Results from the first and second coders were compared using Scott’s *pi*.

### Ethics

This study used publically available television broadcasts and did not include any human participants. As such, ethical approval was not required.

## Results

Unfortunately, 37 minutes of one broadcast (Sweden vs England) were not available due to a temporary power cut during recording. This left 1487 minutes of broadcast in the analysis. Second coding of one randomly selected broadcast revealed a very high level of agreement between coders (*pi* = 0.79, where *pi* = 1 represents exact agreement).

A total of 1846 visual references to alcohol were identified - a mean of 1.24 visual references to alcohol per minute of broadcast. There was substantial variation between broadcasts with mean number of visual references to alcohol per minute varying from 0.85 to 1.65 (Table 2).

**Table 2:** Number (% of column total) of visual references to alcohol in EURO2012 matches

	***Ireland v Croatia***	***France v England***	***Sweden v England***	***England v Ukraine***	***Germany v Greece***	***England v Italy***	***Portugal v Spain***	***Spain v Italy***	***England matches***	***Other matches***	***BBC1 matches***	***ITV1 matches***	***Total***
Location													
Pitch side	135 (74.6)	212 (79.1)	206 (91.6)	229 (81.2)	161 (98.2)	305 (93.6)	229 (97.9)	164 (98.8)	952 (86.5)	689 (92.5)	904 (95.1)	737 (82.3)	1641 (88.9)
Interview boards	11 (6.1)	33 (12.3)	18 (8.0)	29 (10.3)	0	20 (6.1)	4 (1.7)	0	100 (9.1)	15 (2.0)	42 (4.4)	73 (8.2)	115 (6.2)
Commercial breaks	28 (15.5)	20 (7.5)	NA^a^	21 (7.4)	3 (1.8)	NA	NA	NA	41 (3.7)	31 (4.2)	NA	72 (8.0)	72 (3.9)
Other	7 (3.9)	3 (1.1)	1 (0.4)	3 (1.1)	0	1 (0.3)	1 (0.4)	2 (1.2)	8 (0.7)	10 (1.3)	5 (0.5)	13 (1.5)	18 (1.0)
Alcohol type													
Beer	171 (94.5)	260 (97.0)	225 (100)	276 (97.9)	164 (100)	326 (100)	234 (100)	166 (100)	1087 (98.7)	735 (98.7)	951 (100)	871 (97.3)	1822 (98.7)
Cider	6 (3.3)	5 (1.9)	0	3 (1.1)	0	0	0	0	8 (0.7)	6 (0.8)	0	14 (1.6)	14 (0.8)
Spirits	0	0	0	0	0	0	0	0	0	0	0	0	0
Wine	0	0	0	0	0	0	0	0	0	0	0	0	0
Alcopops	4 (2.2)	3 (1.1)	0	3 (1.1)	0	0	0	0	6 (0.5)	4 (0.5)	0	10 (1.1)	10 (0.5)
Broadcast segment													
Pre-match	7 (3.9)	16 (6.0)	1 (0.4)	13 (4.6)	1 (0.6)	35 (10.7)	8 (3.4)	14 (8.4)	65 (5.9)	30 (4.0)	58 (6.1)	37 (4.1)	95 (5.1)
1st half	60 (33.1)	87 (32.4)	89 (39.6)	72 (25.5)	71 (43.3)	109 (33.4)	72 (30.8)	64 (38.6)	357 (32.4)	267 (35.8)	334 (35.1)	290 (32.4)	624 (33.8)
Half-time	0 (0)	16 (6.0)	11 (4.9)	20 (7.1)	12 (7.3)	28 (8.6)	20 (8.5)	8 (4.8)	75 (6.8)	40 (5.4)	67 (7.0)	48 (5.4)	115 (6.2)
2nd half	75 (41.4)	75 (28.0)	81 (36.0)	93 (33.0)	75 (45.7)	88 (27.0)	81 (34.6)	75 (45.2)	337 (30.6)	306 (41.1)	325 (34.2)	318 (35.5)	643 (34.8)
Extra time/penalties	NA^b^	NA	NA	NA	NA	40 (12.3)	46 (19.7)	NA	40 (3.6)	46 (6.2)	86 (9.0)	NA	86 (4.7)
Post-match	11 (6.1)	54 (20.1)	43 (19.1)	63 (22.3)	2 (1.2)	26 (1.24)	7 (3.0)	5 (3.0)	186 (16.9)	25 (3.4)	81 (8.5)	130 (14.5)	211 (11.4)
Commercial breaks	28 (15.5)	20 (7.5)	NA	21 (7.4)	3 (1.8)	NA	NA	NA	41 (3.7)	31 (4.2)	NA	72 (8.0)	72 (3.9)
Total in-play	135 (74.6)	162 (60.4)	170 (75.6)	165 (58.5)	146 (89.0)	228 (69.9)	198 (84.6)	139 (83.7)	725 (65.8)	618 (83.0)	735 (77.3)	608 (67.9)	1343 (72.8)
Total out-of-play	46 (25.4)	106 (39.6)	55 (24.4)	117 (41.5)	18 (11.0)	98 (30.1)	36 (15.4)	27 (16.3)	376 (34.2)	127 (17.0)	216 (22.7)	287 (32.1)	503 (27.3)
Total	181 (100)	268 (100)	225 (100)	282 (100)	164 (100)	326 (100)	234 (100)	166 (100)	1101 (100)	745 (100)	951 (100)	895 (100)	1846 (100)
Mean per minute in-play	1.38	1.72	1.79	1.77	1.54	1.77	1.51	1.48	1.76	1.48	1.63	1.60	1.62
Mean per minute out-of-play	0.72	0.93	1.34	1.21	0.31	1.00	0.43	0.26	1.07	0.46	0.67	0.86	0.76
Mean per minute overall	1.12	1.29	1.65	1.48	1.06	1.44	1.09	0.85	1.45	1.03	1.23	1.25	1.24

^a^NA: not applicable, these matches were broadcast on BBC1, a public service broadcaster, that does not include commercial breaks; ^b^NA: not applicable, these matches did not include extra time or penalties.

There was evidence that the mean number of visual references to alcohol per minute was higher in matches that featured England (m = 1.46, sd = 0.15), than in other matches (m = 1.03, sd = 0.12; t = 4.53, p = 0.004). There was no statistical differences in the mean number of visual references to alcohol in matches broadcast on BBC1 (m = 1.26, sd = 0.36), compared to those broadcast on ITV1 (m = 1.24, sd = 0.19; t = 0.10, p = 0.92).

The majority of visual references to alcohol were pitch-side (88.9% overall). The location of visual references to alcohol varied significantly between matches that did and did not feature England (*χ*^2^(3) = 39.4, p < 0.001), with a higher proportion of references featured on interview boards in England matches. The location of visual references to alcohol also varied between matches broadcast on BBC1 and ITV1 (*χ*^2^(3) = 99.3, p < 0.001). This primarily reflects the absence of commercial breaks on BBC1.

The proportion of visual references to alcohol that related to beer was universally high (98.7%) and did not vary according to whether or not matches featured England (*χ*^2^(2) = 0.04, p = 0.98). Whilst all visual references to alcohol in broadcasts on BBC1 were for beer, those in broadcasts on ITV1 were a little more varied (*χ*^2^(2) = 25.84, p < 0.001); also including references to cider and alcopops.

Overall, almost three-quarters (72.8%) of visual references to alcohol were in-play, although this varied between matches from 58.5% to 89.0%. The proportion of visual reference to alcohol that were in-play was lower in matches that featured England than in matches that did not (*χ*^2^(1) = 65.57, p < 0.001). This reflects differences in location of references seen between matches that did and did not feature England. The proportion of visual references to alcohol that were in-play was also higher in matches broadcast on BBC1 than those broadcast on ITV1 (*χ*^2^(1) = 20.35, p < 0.001). This reflects the absence of commercial breaks on BBC1, driving down the opportunities for visual references out-of-play.

## Discussion

### Statement of principal findings

This is the first analysis of alcohol marketing in a televised international football tournament that the authors are aware of. It is also the first to systematically compare broadcasts on commercial and public service channels, and matches featuring the ‘home’ team with other matches.

A high level of alcohol marketing in EURO2012 matches broadcast on UK television was found, with a mean of 1.24 visual references to alcohol per minute of broadcast. The great majority of these were simple visual representations of brand logos. The frequency of visual references to alcohol was significantly higher in matches featuring England than in other matches. The location of visual references to alcohol also varied significantly between England and other matches, with more references on interview boards in England matches. The frequency of visual references to alcohol did not vary between matches broadcast on the public service and commercial channel. Matches on the commercial channel included more visual references to alcohol in commercial breaks. Whilst the great majority of references were to beer products, more variety in products referred to was seen on the commercial channel than the public service channel.

### Strengths and weaknesses of the study

Previous studies have explored the frequency of alcohol marketing in a range of televised sports [5,15-18]. However, few have focused on football – perhaps the world’s most popular spectator sport – and few have focused on international tournaments. EURO2012 is one of the most prestigious football tournaments in the world. Given the known impact of alcohol marketing on the behaviour of children in particular [1-3], and the worldwide exposure to EURO2012 [12], this study has substantial global public health and policy relevance.

A coding framework was used that has previously been identified as having good inter-rater reliability [14] and this was confirmed in the present study. However, all visual references to alcohol were treated the same and we did not distinguish in terms of the time that visual references were on screen, or the creative power of different references. The power of marketing reflects a combination of impact and exposure. This study focused on frequency of exposure only. Determining the creative impact of different visual references to alcohol would require more detailed, perhaps laboratory-based, analysis. Neither was length of exposure considered. This is because many visual references were on screen for very short periods and we felt it was impractical to record the length of such short exposures accurately.

Although the absolute number of matches included (eight) was small, this represents more than one-third of matches in EURO2012 and is, therefore, a relatively high proportion of eligible matches. The included matches totalled almost 25 hours of broadcasting. The number of matches, and total hours included, represents an improvement on a number of previous, smaller studies [5,14,15]. A stratified, random sampling procedure was also used to select matches for inclusions, reducing the potential for bias.

Previous work, which focused on alcohol marketing in televised English professional club football, found some evidence that the level of marketing found was related to whether or not the competition being contested had title alcohol sponsorship [14]. As all matches in this study were from the same competition, variations in sponsorship between matches were not present, again reducing the potential for bias.

This study focused exclusively on alcohol marketing in television broadcasts of EURO2012 matches. Although this, in itself, is a complex area [5], alcohol marketing associated with sporting events extends beyond television, particularly to online and social media spheres [19-21], and none of this was captured. The extent, nature and context of this marketing is largely unknown and future research should make attempts to explore this further [22].

### Interpretation and implications of findings

The frequency of visual references to alcohol identified here was lower than found in a study of televised English professional club football which used similar methods (and reported a mean of 1.9 references per minute) [14]. Other research has used substantially different methods [5,15,18,23,24], making comparisons difficult. However, one study of rugby league matches broadcast in Australia documented 4062 episodes (of at least 2 s duration) of alcohol marketing during 360 minutes of coverage, representing 11.3 episodes per minute - a nearly ten-fold increase on the frequency found here [15]. A further study from New Zealand including a range of rugby, cricket and tennis matches found a mean of 2.98 visual representations of alcohol per minute across more than 110 hours of broadcasting, although this frequency varied considerably by sport [16]. These very high levels may represent a focus on match-time, but nevertheless highlights the considerable international and inter-sport variability in alcohol marketing in televised sport.

Given the variety of methods used in the literature to measure television alcohol marketing, consensus on the most useful measure is required. Further work could explore which method is most associated with audience recall, as well as behaviour.

Our finding that matches featuring England had significantly more visual references to alcohol per minute than other matches is likely explained by the finding that the distribution of locations of visual references varied significantly between England and other matches. During England matches, a higher proportion of visual references to alcohol were on ‘interview boards’ than in other matches. These floor-to-ceiling boards, covered in sponsor logos, are used as backdrops for pre- and post-match interviews with players, managers and other team staff. England was the only UK team that took part in EURO2012. It is, therefore, understandable that UK broadcasters would be particularly interested in interviews with players and staff from the England team. This is further reinforced by the finding that the proportion of visual references to alcohol that were in-play was smaller in matches featuring England than in other matches – again suggesting a greater focus on out-of-play features.

It is also notable that England matches had higher mean UK-viewing figures than other matches in the sample. This means the higher frequency of visual references to alcohol in England matches resulted in substantially higher population exposure to alcohol marketing during these matches than other matches.

The finding that mean number of visual references to alcohol per minute did not vary between matches broadcast on BBC1 (a non-commercial public service broadcaster) and ITV1 (a commercial free-to-air broadcaster) reinforces how embedded alcohol marketing is in football. The absence of commercial breaks on BBC1 had no impact on the overall burden of alcohol marketing. However, there were differences in the location of visual references to alcohol between matches on BBC1 and ITV1 – with a number of references to alcohol found in commercial breaks on ITV1. This was further reflected in the finding that the proportion of visual references to alcohol that were in-play was lower in matches broadcast on ITV1 compared to BBC1 – highlighting the differing opportunities for marketing on different channels.

EURO2012 did not have a title sponsor. However, *Carlsberg* was one of the main commercial partners of the tournament. This provided significant marketing and sales rights within stadia, and ‘fan-zones’, to the exclusion of all other alcohol brands. This likely explains why the great majority of visual references to alcohol in theincluded matches were for beer.

The Portman Group – the UK alcohol industry’s ‘responsibility body’ – has recently produced a new code-of-practice on alcohol event sponsorship, which came into force in January 2014 [8]. Whilst this reinforces that alcohol sponsorship should not be used in child-specific events and should not associate alcohol with socially successful or anti-social behaviour, it does nothing to restrict the sheer volume of alcohol marketing found in relation to sponsorship of events such as EURO2012.

There is some evidence that frequent exposure to simple alcohol marketing cues, such as those identified in this study, has a greater effect on familiarity and positive brand attitudes than more traditional advertising [25]. It is possible that industry focuses self-regulatory codes-of-practice on marketing content, rather than exposure, as this gives a superficial appearance of responsibility at the same time as providing greater lee-way for exploitation of frequent exposure effects [26]. The focus of self-regulatory codes-of-practice on content, rather than exposure has been identified by others as a weakness of this approach to regulation [3,27]. The propensity of the worldwide alcohol industry to revise codes-of-practice by removing commonly violated clauses, rather than increasing enforcement, has also been previously noted [28].

Furthermore, whilst the Portman Group’s newly introduced code-of-practice on event sponsorship states that sponsorship of children-specific events is not appropriate, it does not restrict sponsorship of sporting events such as EURO2012 that are highly attractive to individuals of all ages [8]. The apparent ease with which the alcohol industry - in the UK and elsewhere - undermines self-regulatory codes that restrict marketing to those above the minimum drinking age has been previously described [28-31].

The impact of alcohol marketing on children has been clearly demonstrated at systematic review level [1-3]. However, the specific effect of alcohol marketing in televised sport on alcohol consumption in both adults and children is not clear and requires further investigation. Previous research on tobacco sponsorship of televised sporting events found that it impacted on children’s recall, preference and self identification with relevant brands; as well as tobacco use [32].

## Conclusion

More than one visual reference to alcohol per minute was identified in a representative sample of matches from EURO2012 broadcast in the UK. This did not vary between matches broadcast on public service and commercial channels highlighting the embedded nature of alcohol marketing in televised sports. A higher frequency of visual references to alcohol was found in matches featuring England than in other matches. This appears to reflect a particularly high volume of marketing found around player interviews. As these matches attracted higher UK audiences, this led to greater overall population exposure to alcohol marketing. Current self-regulatory codes-of-practice appear to be inadequate to control the type of marketing found in televised sport in the UK.

## Competing interests

The authors declare that they have no competing interests.

## Authors’ contributions

JA conceived the idea for the research. JA, JC and MW developed the methods. JC drafted the protocol and collected the data. JA and JC analysed the data. JA, JC & MW interpreted the results. JA drafted the manuscript. All authors critically reviewed the manuscript. All authors read and approved the final manuscript.

## Pre-publication history

The pre-publication history for this paper can be accessed here:

http://www.biomedcentral.com/1471-2458/14/473/prepub
